# Androgen Receptor and Poly(ADP-ribose) Glycohydrolase Inhibition Increases Efficiency of Androgen Ablation in Prostate Cancer Cells

**DOI:** 10.1038/s41598-020-60849-y

**Published:** 2020-03-02

**Authors:** Manqi Zhang, Yanhao Lai, Judy L. Vasquez, Dominic I. James, Kate M. Smith, Ian D. Waddell, Donald J. Ogilvie, Yuan Liu, Irina U. Agoulnik

**Affiliations:** 1 Biochemistry Ph.D. Program, Florida International University, Miami, FL USA; 2 Department of Chemistry and Biochemistry, College of Arts, Sciences and Education, Florida International University, Miami, FL USA; 3 Biomolecular Sciences Institute, Florida International University, Miami, FL USA; 4 Department of Human and Molecular Genetics, Herbert Wertheim College of Medicine, Florida International University, Miami, FL USA; 5 Cancer Research UK Manchester Institute, The University of Manchester, Alderley Park, SK104TG UK; 6 Present Address: CRL, Chesterford Research Park, CB10 1XL Alderley Park, UK; 7 Department of Molecular and Cellular Biology, Baylor College of Medicine, Houston, TX USA

**Keywords:** DNA, Prostate cancer

## Abstract

There is mounting evidence of androgen receptor signaling inducing genome instability and changing DNA repair capacity in prostate cancer cells. Expression of genes associated with base excision repair (BER) is increased with prostate cancer progression and correlates with poor prognosis. Poly(ADP-ribose) polymerase (PARP) and poly(ADP-ribose) glycohydrolase (PARG) are key enzymes in BER that elongate and degrade PAR polymers on target proteins. While PARP inhibitors have been tested in clinical trials and are a promising therapy for prostate cancer patients with TMPRSS2-ERG fusions and mutations in DNA repair genes, PARG inhibitors have not been evaluated. We show that PARG is a direct androgen receptor (AR) target gene. AR is recruited to the PARG locus and induces PARG expression. Androgen ablation combined with PARG inhibition synergistically reduces BER capacity in independently derived LNCaP and LAPC4 prostate cancer cell lines. A combination of PARG inhibition with androgen ablation or with the DNA damaging drug, temozolomide, significantly reduces cellular proliferation and increases DNA damage. PARG inhibition alters AR transcriptional output without changing AR protein levels. Thus, AR and PARG are engaged in reciprocal regulation suggesting that the success of androgen ablation therapy can be enhanced by PARG inhibition in prostate cancer patients.

## Introduction

Late stage prostate cancers are treated with radiation and other cytotoxic therapies. It was observed that androgen ablation sensitizes prostate tumors to radiation and chemotherapy in prostate cancer patients^1–3^. Prostate tumors with high androgen receptor (AR) transcriptional output have increased expression of DNA repair genes in general, and base excision repair (BER) associated proteins in particular^4,5^. Conversely, AR upregulates pathways in prostate cancer that increase genomic instability, such as the TMPRSS2-ERG gene fusion, which is present in a significant part of advanced prostate cancers^5,6^. Importantly, inhibition of poly(ADP-ribose) polymerase 1 (PARP1) significantly increases levels of DNA damage in such tumors^7^ and is currently being tested in clinical trials in metastatic castration resistant prostate cancer (CRPC) with somatic or germline mutations in DNA repair genes. The PARP inhibitors rucaparib (NCT02952534, NCT03533946 and NCT03413995) and olaparib (NCT02316197, NCT03012321, NCT03787680, NCT03432897 and others) have been tested in clinical trials and were granted breakthrough designation by the U.S. Food and Drug Administration (FDA) for metastatic CRPC.

Poly(ADP-ribose) glycohydrolase (PARG) and PARP1 are key enzymes required for DNA repair and maintenance of genomic stability. Poly ADP-ribose (PAR) is a heterogeneous branched polymer of ADP-ribose that is attached to proteins in response to various stimuli by a dynamic process called PARylation. PARylation regulates DNA damage detection and repair as PAR acts as a loading platform to recruit a variety of DNA repair factors, in a non-covalent fashion, to the DNA lesions. Multiple PARPs synthesize PAR polymers, while the bulk of polymeric PAR is hydrolyzed by PARG via exo- and endo-glycosylase activities that generate mono (ADP-ribose) residues^8^. These mono (ADP-ribose) residues can be further hydrolyzed by ARH1, ARH3, and macrodomain proteins^9^. PARylation of PARP1 and other DNA repair proteins is required for efficient DNA repair by multiple mechanisms, including BER^10–12^. Single-nucleotide BER is mediated by the functional interactions of apurinic/pyrimidinic endonuclease 1 (APE1), PARP1, DNA polymerase β (Pol β), X-ray repair cross-complementing protein 1 (XRCC1), and DNA ligase III (LIG III). DNA glycosylase and APE1 remove DNA lesions and create a single-strand DNA break (SSB) which stimulates the recruitment and activation of PARP1. PARP1 then PARylates itself, histones, and other DNA damage repair proteins^13^ relaxing the chromatin fiber and increasing the accessibility of damage sites to repair enzymes and cofactors such as XRCC1^14–17^, LIG III, and Pol β^18,19^. PARP1 then interacts with XRCC1 and activates LIG III^20^. PARG mediated dePARylation of PARP1 and other proteins is critical for maintaining multiple rounds of efficient DNA damage repair^21^. When PARylated, the AR transcriptional coregulator KDM4D is also a PARG substrate^22^. KDM4D interacts directly with AR^23^ and other steroid receptors^22^, and PARylation inhibits its ability to stimulate the transcriptional activity of steroid receptors in a promoter dependent manner^22^. A thorough evaluation of PARG function in prostate cancer was hampered by a lack of stable and bioavailable inhibitors. Recently, we synthesized and characterized a number of novel and specific PARG inhibitors, including PDD00017272^24^. In this report, we tested whether androgen deprivation synergized with PARG inhibition to suppress prostate cancer cell growth. We found that inhibition of PARG synergized with androgen deprivation to reduce BER capacity and inhibit cellular proliferation and viability in multiple prostate cancer cell lines. Similar to retinoic acid receptor (RAR), PARG inhibition altered the AR transcription in a promoter-dependent manner. Treatment with a PARG inhibitor increased levels of the DNA damage-associated γ-H2A.X histone variant. Reduction in BER capacity was due, in part, to increased levels of PARP1 PARylation. In summary, we show that androgen receptor signaling stimulates DNA repair in part through increasing basal levels of PARG expression, suggesting a novel therapeutic target for prostate cancer patients with advanced disease.

## Results

### AR stimulates PARG expression

**Figure 1:**
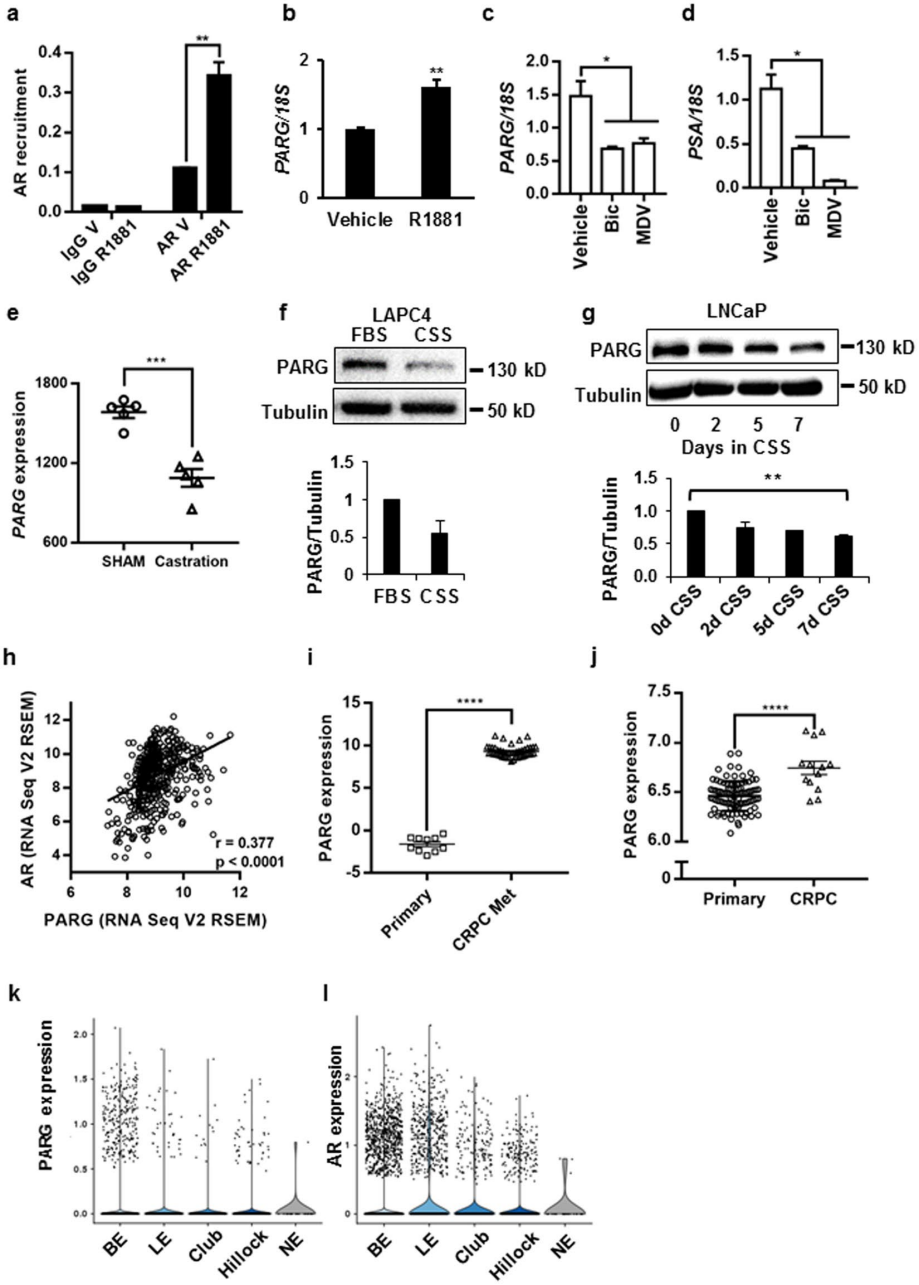
AR regulation of PARG expression in independent models. (**a)** LNCaP cells were grown in medium supplemented with CSS and treated with ethanol vehicle or 10 nM of R1881 for 18 hours. AR recruitment to the PARG locus was compared by a ChIP Assay. Each bar represents an average of 3 biological replicates, p = 0.0012. The experiment was repeated 4 times. (**b**) RNA was extracted from LNCaP cells treated in parallel with (**a**), reverse transcribed, and used for qPCR to compare PARG mRNA levels. Expression was normalized to 18S. p = 0.00650. Each bar is an average of 3 biological replicates, the experiment was performed 4 times. (**c**,**d**) LNCaP cells were grown in medium supplemented with 10% FBS and treated with DMSO, 10^–5^ M of bicalutamide (Bic) or MDV3100 for 72 hours. RNA was isolated, reverse transcribed, and analyzed for PARG (**c**) and PSA (**d**) expression using 18S as control. Each bar is an average of 3 biological replicates. Experiment was repeated 3 times. (**e**) Values for PARG expression in LuCaP35 PDX samples grown in intact (N = 5) and castrated (N = 5) mice were exported from GSE33316 and averaged (p = 0.00025). (**f**) Top: PARG expression in LAPC4 cells grown in FBS or CSS supplemented media for 48 hours. Representative western blot shown. Bottom: average quantification of PARG protein levels normalized for Tubulin from 3 independent experiments. **(g)** LNCaP cells grown in FBS or CSS for 2, 5, and 7 days. Experiments f-g were repeated 3 times and representative blots are shown. **(h)** Correlation between PARG expression and AR expression in 499 prostate adenocarcinoma samples (TCGA, Provisional). **(i)** PARG expression in samples of 10 primary prostate tumors and 45 metastatic CRPC tumors in data set GSE74367. **(j)** PARG expression in 94 primary prostate tumors and 13 CRPC prostate tumors in data set GSE70770. **(k**,**l)** The violin plots show the expression of PARG (k) and AR (l) in basal epithelia (BE), luminal epithelia (LE), club epithelia (Club), Hillock epithelia (Hillock), and neuroendocrine (NE) of human prostate cells (GSE120716, http://strandlab.net/analysis.php). (*p ≤ 0.05; **p ≤ 0.01; ***p ≤ 0.001; ****p ≤ 0.0001).

AR and its pioneer factor FoxA1 chromatin recruitment in LNCaP cells has been reported by several groups^25,26^. As seen in Supplementary Fig. 1^25^, AR was highly recruited to multiple sites in the PARG locus in both ligand-dependent and -independent manners, concomitantly with the AR pioneer factor FoxA1. Using a chromatin immunoprecipitation (ChIP) assay, we confirmed both androgen-dependent and -independent recruitment of AR to the FoxA1 recruitment sites within the PARG locus in LNCaP cells (Fig. 1a). PARG mRNA expression was upregulated in the presence of the synthetic androgen methyltrienalone (R1881) in LNCaP cells (Fig. 1b) and with DHT treatment in the independently derived LNCaP and LAPC4 prostate cancer cell lines (Supplementary Fig. 2a,b). Conversely, three-day treatment with bicalutamide or MDV3100 reduced expression of PARG in LNCaP cells (Fig. 1c). Interestingly, bicalutamide inhibited PARG to a similar extent as the direct AR target gene PSA, while MDV3100 was more efficient in inhibiting PSA than PARG (Fig. 1d) suggesting differential regulation of these target genes. Significant reduction of PARG expression in response to bicalutamide and MDV3100 treatment was also observed in an independent gene expression dataset GSE62474 (Supplementary Fig. 2c). Consistently, in primary prostate cancer LuCaP35 xenografts tumors, intratumoral PARG expression declined in males 4 weeks after castration when compared to tumors from SHAM operated animals which underwent all surgical procedures except removal of the testicles [GSE33316] (Fig. 1e). Androgen withdrawal decreased PARG protein levels in both LNCaP and LAPC4 cell lines (Fig. 1f,g). Consistent with our observed PARG induction by AR in prostate cancer cell lines, we noted a highly significant correlation between AR and PARG expression in prostate tumors^27,28^ (Fig. 1h). AR levels and activity are significantly elevated in CRPC^29,30^. Accordingly, we observed significant increases in PARG expression with CRPC compared to primary cancer in the data sets GSE74367 and GSE70770 (Fig. 1i,j)^31,32^. Based on the data generated by single cell sequencing of the normal human prostate, there was a significant overlap in AR and PARG expression in prostate epithelial compartments, further confirming the functional interaction between AR and PARG (Fig. 1kl)^33^.

### AR-V7 does not regulate PARG expression

**Figure 2:**
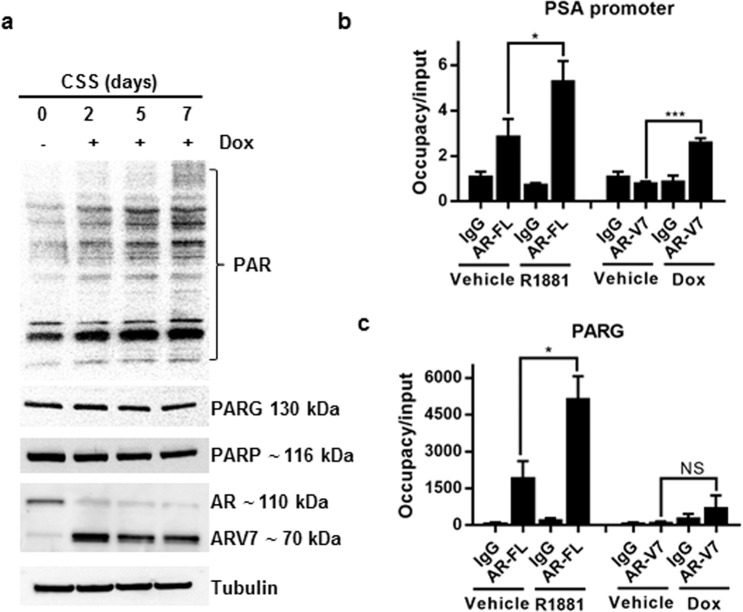
AR-V7 does not regulate PARG levels or activity. (**a**) LNCaP^AR-V7/pHage^ cells were plated in medium with 10% CSS. Cells were treated with 50 ng/ml Dox for 0, 2, 5, and 7 days. Protein levels of PAR, PARG, PARP-1, AR, AR-V7, and Tubulin were tested by Western blotting. The experiment was repeated 3 times and a representative blot is shown. (**b**,**c**) LNCaP^AR-V7/pHage^ cells were placed in medium with 10% CSS for 24 hours and treated with 50 ng/ml Dox or 10 nM R1881 overnight as indicated. Recruitment of AR-FL and AR-V7 to PSA promoter (**b**) and PARG (**c**) were measured by ChIP-qPCR using Igg as control. (*p ≤ 0.05; ***p ≤ 0.001). In panels b-c, each bar represents an average of 3 biological replicates, the experiment was repeated 3 times.

In patients, the develpoment of resistance to castration therapy is frequently associated with the elevated expression of AR splice variants which lack the ligand binding domain and activate an overlapping but distinct set of target genes^34^. One of the most common AR splice variants associated with poor prognosis is AR-V7^35,36^. Using the previously described LNCaP^AR-V7/pLenti^ cell line^37^, we investigated whether AR-V7 regulates PARG expression similarly to full length AR. As seen from Fig. 2a, doxycycline significantly induced the expression of AR-V7. AR-V7 induction did not increase either PARG or PARP protein level and correspondingly did not downregulate levels of PAR in LNCaP cells (Fig. 2a). Analysis of full length AR (AR-FL) and AR-V7 recruitment to DNA showed that while both AR variants were recruited to the PSA promoter, only full length AR was recruited to the PARG locus (Fig. 2b,c).

### Downregulation of PARG leads to PAR accumulation

**Figure 3:**
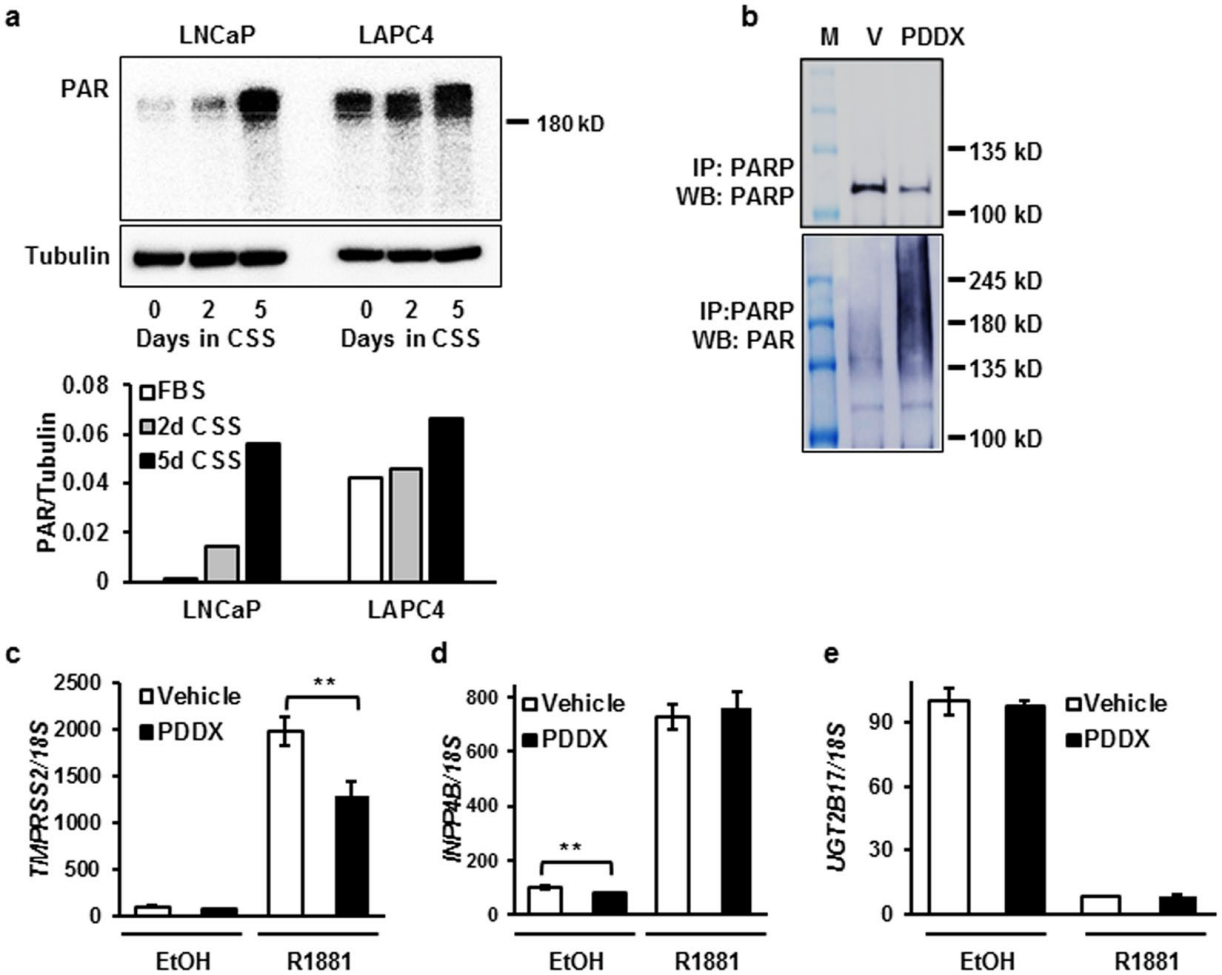
PDDX treatment causes an accumulation of PAR in cells. (**a**) PAR accumulation in LNCaP and LAPC4 cells during androgen withdrawal for 2 and 5 days analyzed by western blot. Quantification of PAR normalized to tubulin is shown under the western blot image. (**b**) LNCaP cells were treated with either vehicle or PDDX inhibitor for 48 hours. PARP1 was immunoprecipitated and the eluates were probed with PARP1 (top) or PAR antibody (bottom). **(c–e)** LNCaP cells were grown in medium supplemented with CSS and treated with vehicle, 0.1 nM R1881, 1 µM PDDX, or 0.1 nM R1881 and 1 µM PDDX for 48 hours as indicated. RNA was analyzed for expression of AR target genes TMPRSS2 (**c**), INPP4B (**d**) and UGT2B17 (**e**) (**p ≤ 0.01). All experiments were performed at least 3 times. In panels c-e, each bar is an average of 3 biological replicates.

Androgen withdrawal led to a decline in PARG expression (Fig. 1f,g), and consequentially, the accumulation of high molecular weight PAR in both LNCaP and LAPC4 cell lines (Fig. 3a). As expected, DHT was able to reduce PAR accumulation caused by androgen withdrawal to levels observed in medium supplemented with FBS (Supplementary Fig. 3a). Since PARP1 is a known target of PARG activity, we tested whether PARG inhibition by PDD00017272 (henceforth referred to as PDDX) increases the PARylation levels of PARP1. Indeed, PARylation of PARP1 was significantly increased by the inhibition of PARG activity (Fig. 3b). In previous reports it was shown that PARG dePARylates the AR coactivator KDM4D, which physically interacts with AR and potentiates AR activity in a promoter-dependent manner^23^. We next investigated whether PARG inhibition affects AR transcriptional activity. As seen from Fig. 3c, ligand-dependent induction of the AR target gene TMPRSS2 was significantly reduced by the PARG inhibitor. However, the AR-mediated induction of INPP4B (Fig. 3d) and repression of UGT2B17 (Fig. 3e) were not affected. Thus, similar to the retinoic acid receptor^22^, PARG activity is required for the transcriptional regulation of a subset of AR target genes.

### PARG inhibition synergizes with androgen ablation to inhibit BER

**Figure 4:**
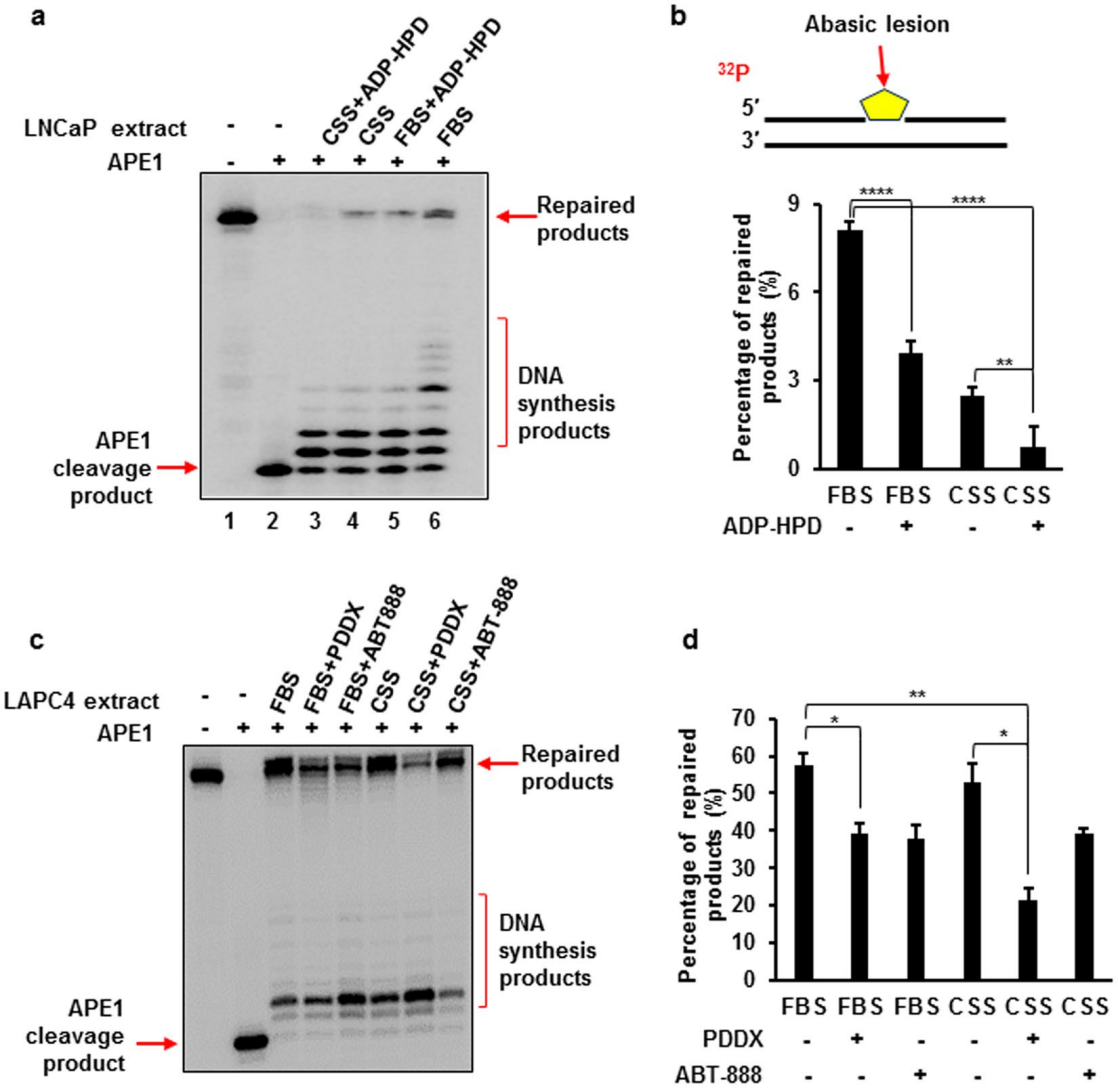
Androgen Receptor and PARG activities are required for optimal BER capacity in androgen-dependent prostate cancer cells. BER capacity was measured by incubating a random DNA sequence substrate containing an abasic site (a THF residue) with cell extracts from LNCaP **(a**,**b)** and LAPC4 **(c**,**d)** cells under the conditions described in the Materials and Methods. Lane 1 is the untreated substrate. Lane 2 corresponds to substrate treated with 1 nM APE1. All subsequent lanes correspond to reaction mixtures supplemented with extracts of cells grown in FBS or CSS and treated as indicated. Substrate was ^32^P-labeled at the 5′-end of the damaged strand as shown in 3b. The signal was detected with phosphoimager, and the intensity of the ligated product was quantified by the Quantity One software. Signal and standard deviation were calculated and presented as a bar graph for LNCaP (**b**) and LAPC4 (**d**). The experiments were repeated three times. Representative gels are shown. (*p ≤ 0.05; **p ≤ 0.01; ****p ≤ 0.0001). Experiments in a and c were performed at least 3 times. Calculations in b and d were done for 3 independent experiments.

AR induces the expression of multiple DNA repair genes, including PARG, which are essential for multiple steps in BER^4^. We therefore tested whether androgen deprivation synergizes with the inhibition of PARG in reducing BER capacity. FBS contains levels of steroids sufficient to fully activate AR transcriptional activity. To simulate a castration-like condition, we supplemented growth medium with charcoal stripped serum (CSS) in which steroid hormones and some growth factors and cytokines which activate AR are depleted. In these conditions, AR-dependent expression of DNA repair related genes is significantly decreased^4^. Using a previously developed method for quantifying BER capacity^38^, we compared the levels of BER using a synthetic BER substrate mixed with cell lysates. The lysates were prepared from LNCaP or LAPC4 cells grown in FBS or CSS supplemented medium and treated either with a PARG or PARP1 inhibitor or vehicle control. As indicated by the intensity of the band representing ligated product, the highest level of BER capacity was observed in the lysate of cells grown in FBS medium (Fig. 4a,c). In LNCaP cells, BER capacity decreased by 68% when cells were grown in androgen depleted medium (Fig. 4a,b). Similar results were obtained with PDDX (data not shown). Treatment with a PARG inhibitor during androgen deprivation decreased BER capacity of the lysates by 90%, when compared with cells maintained in FBS containing medium (FBS vs CSS + ADP-HPD) (Fig. 4b). In LAPC4 cells, the inhibition of PARG in FBS supplemented medium decreased BER capacity by 31% (FBS vs FBS + PDDX). Androgen deprivation decreased BER capacity by 7% compared with FBS, however, the combination of androgen deprivation and PARG inhibition reduced BER capacity by 62% (FBS vs CSS + PDDX) (Fig. 4d). It should be noted that only the fully ligated products labeled as “Repaired products” in the Fig. 4a,c were used for quantification in Fig. 4b,d. This is because the repaired products must be ligated in order to represent the overall BER capacity in the cells.

### Combination of androgen deprivation and PARG inhibition causes accumulation of DNA damage

**Figure 5:**
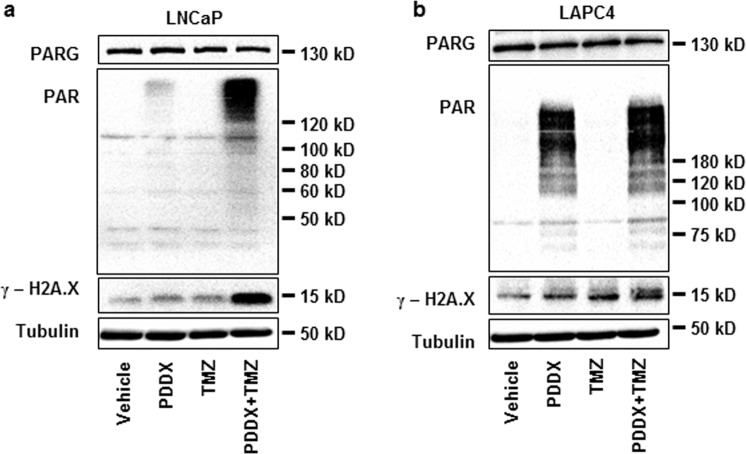
Inhibition of PARG in androgen-depleted medium causes DNA damage. (**a**) LNCaP cells were plated in FBS medium. The following day medium was replaced with one supplemented with CSS and treated with vehicle control, 1 µM of PDDX, 100 µM temozolomide, or both, for 48 hours. Representative western blot shown. (**b**) LAPC4 cells were plated in FBS medium containing 1 nM R1881. The following day medium was replaced to one with CSS and cells were treated as in (**a**) for 48 hours. Proteins were extracted and levels of PARG, PAR, γ-H2A.X, and Tubulin were compared using western blotting. Experiments shown in this figure were performed 3 times.

BER inhibition leads to the accumulation of SSBs eventually resulting in double-strand breaks (DSB), which can be evaluated by the accumulation of the phosphorylated H2A histone variant, γ-H2A.X^39^. We tested whether a reduced BER capacity to repair SSBs caused by the alkylating agent, temozolomide (TMZ), would lead to an increase in γ-H2A.X protein level. It has been previously shown that prolonged incubation of LNCaP cells in CSS leads to an accumulation of γ-H2A.X^40^. Similarly, we showed a very low level of γ-H2A.X in LNCaP and LAPC4 cells grown in medium supplemented with FBS, a small but significant increase after 48-hour incubation in CSS supplemented medium and a further increase in cells growing in medium supplemented with CSS and treated with PDDX for 48 hours (Supplementary Fig. 3b,c). While short term treatment with temozolomide or PDDX alone caused a minimal increase in γ-H2A.X, the combination of these treatments significantly upregulated levels of this histone variant in both prostate cancer cell lines (Fig. 5a,b).

### PARG inhibition synergizes with androgen deprivation and temozolomide treatment to inhibit prostate cancer proliferation

**Figure 6:**
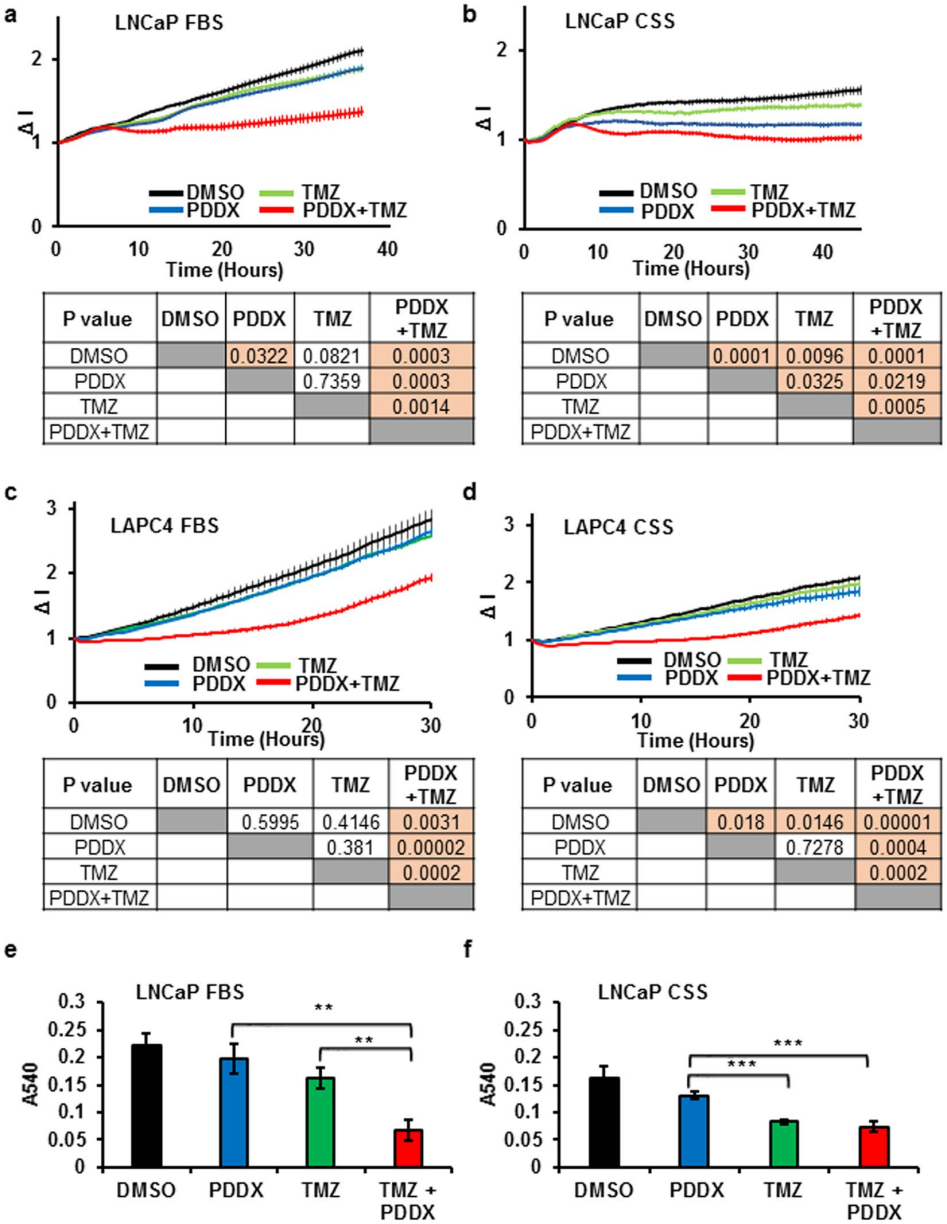
PARG activity is essential for prostate cancer cell proliferation and survival during BER challenge. LNCaP cells were plated onto E-plates in media containing FBS (**a**) or CSS (**b**) and allowed to adhere overnight. The following day cells were treated with either DMSO vehicle, 1 µM of PDDX, 100 µM temozolomide, or a combination of 1 µM PDDX and 100 µM temozolomide. Cell proliferation during the next 40 hours was monitored using the RTCA system. (**c**,**d**) LAPC4 cells were plated into E-plates in the presence of media containing FBS + R1881 (**c**) or CSS (**d**) and allowed to adhere overnight. The following day cells were treated with the same concentration of compounds as LNCaP cells in (**a**,**b)**. Cell proliferation during the next 30 hours of treatment was monitored using the RTCA system. For figures (**a**–**d**), p values for the final time point are shown under the graph. Each point on the panels a-d represents an average of 4 biological replicates and standard error of the mean. The experiments were performed 3 times. (**e**,**f**) LNCaP cells were plated in FBS medium (**e**) or CSS medium (**f**) at a density of 2 × 10^3^ cells per/well. Cells were treated with DMSO vehicle, 1 µM PDDX, 100 µM temozolomide, or a combination of 1 µM PDDX and 100 µM temozolomide for 48 hours. Following treatment, cell viability was determined using the MTT assay. (**p ≤ 0.01; ***p ≤ 0.001). Each bar is an average of 4 biological replicates, experiments were performed 3 times.

To test whether the inhibition of BER and accumulation of γ-H2A.X due to PARG inhibition affected cell proliferation, we compared the effects of temozolomide and PDDX, alone or in combination, and in hormone replete and depleted media. LNCaP cells were only slightly inhibited by either of these treatments in FBS. The combination of temozolomide and PDDX resulted in almost complete growth inhibition as shown by the decreased slope of the curve (Fig. 6a). As expected, LNCaP cells grew slower in CSS medium and proliferation was strongly inhibited by PDDX alone. Strikingly, cell proliferation was abolished by the combined treatment of temozolomide, PDDX and androgen deprivation (Fig. 6b). Neither PDDX nor temozolomide alone strongly inhibited the proliferation of LAPC4 cells in FBS or CSS supplemented media. However, the combination had a profound effect on cellular proliferation in both cases (Fig. 6c,d). Furthermore, an MTT assay showed that the combination of PDDX and temozolomide significantly reduced LNCaP cell viability in both FBS (p ≤ 0.007) and CSS medium (p ≤ 0.001) (Fig. 6e,f).

## Discussion

Prostate cancer progression to metastatic disease is associated with increased AR expression and activity. Thus, the standard-of-care treatment for disease that has spread beyond the prostate capsule is castration therapy. Despite the initial efficacy of this treatment, resistance always develops with the emergence of CRPC^41^. Most CRPCs are characterized by further increases in AR levels and transcriptional output, including the expression of BER-associated genes^5,42^. Importantly, the high expression of DNA repair gene signatures correlates with recurrence, metastases, and poor survival^5^.

Two important enzymes in the BER pathway are PARG and PARP1. While a number of clinical trials are ongoing to evaluate PARP1 inhibition as a prostate cancer therapy, PARG inhibition as a therapeutic strategy has not yet been tested. The value of PARG as a therapeutic target was tested previously in knockdown experiments. PARG knockdown sensitized human cancer cells to radiation and chemotherapies and lead to cell death in breast^43^, colon^44^, pancreatic^45^, ovarian^46^, and glioblastoma cancer cell lines^47^. Treatment with DNA damaging drugs caused up to four times greater cell death in PARG null cells than in WT cells^48^. Strikingly, this is not the case in nonmalignant tissues as PARG inhibitors have a neuroprotective effect, attenuate renal injury, and prevent inflammation after ischemia-reperfusion^49^. PARG inhibitors have also been shown to ameliorate drug-induced hepatotoxicity, cardiotoxicity, and nephrotoxicity^11^. In this study, we investigated the reciprocal regulation between PARG and AR signaling pathways. Mining publicly available AR cistromes, we discovered that AR is recruited to the PARG locus in LNCaP (GSM759659)^50^, C4-2 (GSM1586659)^51^, and VCaP (GSM1328958)^52^ prostate cancer cell lines and in prostate cancer cells from human patients (GSM1358412)^53^. We confirmed AR recruitment to the PARG intron using a ChIP assay in LNCaP cells. Furthermore, AR regulation of PARG expression was confirmed by quantitative RT-PCR and western blotting in two independently derived prostate cancer cell lines (Fig. 1). In LuCaP35 xenograft tumors (GDS4120)^54^, significantly lower PARG mRNA levels were expressed in castrated mice when compared with sham operated animals, confirming an androgen requirement for optimal PARG expression. In the normal human prostate, AR and PARG are expressed in the same types of cells. In human prostate tumors, an AR and PARG functional interaction is supported by highly significant positive Spearman correlations between AR and PARG mRNA levels: r = 0.38, p = 1.00e-17^55^, r = 0.33, p = 5.30e-9^42^, r = 0.41, p = 4.15e-21 (TCGA, Provisional). With prostate cancer progression to CRPC, AR levels and transcriptional outputs are significantly increased with a corresponding elevation of PARG expression (Fig. 1i,j). Notably, the AR splice variant AR-V7 was not recruited to the PARG locus, and ARV7 protein expression did not induce PARG expression (Fig. 2). Consistent with AR induction of PARG levels, growing cells in medium supplemented with CSS decreased the levels of PARG protein (Fig. 1f,g) and increased high molecular weight PAR accumulation (Fig. 3a). PARG substrates include multiple proteins such as histones, DNA polymerases and ligases, XRCC1, p53, Fos, PARP1 and the KDM4 family of androgen receptor coactivators^22,23,56^. Extensive PARylation strongly inhibits PARP1 activity^57^ and alters KDM4D co-activator potential^22^. To test the role of PARG activity in BER and AR signaling we used two PARG specific inhibitors ADP-HPD and PDDX. PDDX is a novel PARG inhibitor which is both specific and bioavailable (Supplementary Fig. 4) that was successfully used in a number of *in vivo* models to inhibit PARG^58,59^. Treatment with PARG inhibitors led to significant increases in the PARylation of PARP1 (Fig. 3b) and changes in AR transcriptional activity in a promoter specific manner (Fig. 3c–e). While androgen ablation leads to decreased expression of PARG, expression is not completely abolished due to the high basal levels of expression (Fig. 1). Some PARG expression always persists amenable to PARG inhibitor treatment. Pharmacological inhibition of residual PARG increases PARylation of PARP1 inhibiting its activity (Fig. 3) and that of other BER-associated proteins. Thus, combination of androgen ablation and PARG inhibition synergizes to reduce BER capacity in androgen dependent prostate cancer cells (Fig. 4). Importantly, we did not observe synergism between androgen ablation and PARP1 inhibition (Fig. 4), likely due to the existence of multiple functional homologues of PARP1 and the lack of androgen regulation of PARP1 expression.

Temozolomide is an alkylating agent that requires functional BER for DNA damage repair and maintenance of cell viability, suggesting a potential synergy between temozolomide treatment and inhibition of PARG^60^ and PARP1^61^. We show that the combination of PARG inhibition, which decreased BER capacity, along with the treatment of temozolomide led to the accumulation of SSB that were subsequently converted to DSBs. This then resulted in the accumulation of γ-H2A.X (Fig. 5). Accumulation of DNA damage in PDDX-temozolomide treated cell lines led to the reduced proliferation and viability of LNCaP and LAPC4 cell lines (Fig. 6). Remarkably, the most significant reduction in proliferation and viability after PDDX-TMZ treatment is observed in androgen depleted conditions, due in part to reduced androgen stimulation of PARG expression and other DNA repair-related proteins^4^. Relatively mild changes in γ-H2A.X and cellular proliferation in cells treated with PDDX alone (Supplementary Fig. 3b,c and Fig. 5) underscore the low toxicity of the PARG inhibitor^59^.

The majority of prostate cancers bear one or more somatic mutations such as the TMPRSS2-ERG fusion, c-Myc overexpression, p53 and Rb mutations, and others which increase genomic instability^62^. Accordingly, somatic and germ line mutations in DNA repair genes, such as BRCA1 and BRCA2^63^, or replication factors^58^, as well as a reduction in DNA repair gene expression due to androgen ablation render tumors vulnerable to PARG inhibitors. This presents a therapeutic opportunity for exploring PARG inhibitors as a supplemental therapy to prostate cancer therapies such as castration, chemotherapy, and radiation. Castration therapies are standard-of-care for men with disseminated prostate cancer. These men are now undergoing clinical trials for treatment with PARP1 inhibitors. While PARP1 levels are not regulated by AR, PARG inhibition has a potential to synergize with castration therapy and be more effective in reducing cancer burden in men with advanced prostate cancer.

We have demonstrated that PARG inhibition can robustly strengthen the response to androgen deprivation and increase DNA damage in prostate cancer cells by reducing BER capacity. Future studies using *in vivo* models are needed to assess the treatment toxicity in non-malignant tissues and efficacy in combination therapies.

## Materials and Methods

### Cell culture

LNCaP and LAPC4 were purchased from American Type Culture Collection (ATCC) and maintained under ATCC-recommended conditions. Fetal Bovine Serum (FBS) and Charcoal Stripped Serum (CSS) were purchased from Sigma-Aldrich (St. Louis, MO). LNCaP^AR-V7/pHAGE^ maintenance was described previously^37^. Tetracycline-screened FBS (TET FBS) was purchased from GE Healthcare (Chicago, IL) and doxycycline from Thermo Fisher Scientific (Manassas, VA). PDD00017272 (referred to as PDDX elsewhere in the manuscript was synthesized at Cancer Research UK Manchester Institute (compound 34 f)^24^. The ammonium salt of ADP-HPD dehydrate was purchased from Calbiochem (San Diego, CA). ABT-888 (veliparib), bicalutamide (Casodex), MDV3100 (enzalutamide), and temozolomide were purchased from Selleckchem (Houston, TX) and dissolved in dimethyl sulfoxide (DMSO). R1881 (Perkin Elmer, Waltham, MA) was dissolved in ethanol (Sigma Aldrich, Milwaukee, WI). DHT and E2 were purchased from Sigma Aldrich (Milwaukee, WI) and dissolved in ethanol.

### Chromatin immunoprecipitation (ChIP) assay

LNCaP cells were plated on a 10 cm plate in 10% FBS at a density of 10^6^ cells/plate and allowed to attach overnight. Cells were then washed with serum free RPMI1640 and medium containing 10% CSS was added. Forty-eight hours later 1 nM of R1881 was added for the indicated period of time and a CHIP assay was performed as previously described^64^. Briefly, formaldehyde was added to media in plates to a concentration of 2% and biomolecules were crosslinked for 30 min at 37 ° C. Cells were rinsed and harvested in PBS and their DNA sheared using Bioruptor Twin sonicator (Diagenode, Denville, NJ). Soluble cellular fractions were divided into 4 equal parts. One part was used as input control and 3 others were used for immunoadsorption with the following antibodies: AR antibody (#06–680, Millipore, Temecula, CA), AR-V7 specific antibody (#68492, Cell signaling, Danvers, MA), Igg antibody (#sc-2028, Santa Cruz, Dallas, TX). Co-immunoprecipitated DNA was recovered by reverse crosslinking and DNA purification using PCR purification kit (QIAGEN, Germantown, MD) The abundance of co-immunoprecipitated DNA from specific loci was compared with quantitative PCR (LightCycler, Roche, Indianapolis, IN) using primers and probes described in Supplemental Table 1.Three biological replicates were used for each variable and the experiments were performed three times. The bars represent average and standard errors. The student t-test was used to test for inequality of means from two independent variables and p value less than 0.05 was considered significant.

### Gene expression analysis

RNA was prepared using TRI Reagent (Molecular Research Center, Cincinnati, OH) and cDNA was prepared using the Verso cDNA Kit (Life Technologies Corporation, Carlsbad, CA). Gene expression was analyzed on a Lightcycler 480 II (Roche, Indianapolis, IN) using LightCycler® 480 Probe Master mix (Roche, Indianapolis, IN) and primers and probes described in Supplemental Table 1. Gene expression was normalized to 18S RNA.

### Western blot analysis

Twenty-five µg aliquots of protein were resolved using a 10% polyacrylamide gel electrophoresis (PAGE) and transferred onto nitrocellulose membrane. Membranes were blocked with 5% BSA in TBS [50 mM Tris-HCl pH 7.5, 150 mM NaCl] at room temperature for one hour and incubated overnight at 4 °C with primary antibodies for Tubulin (#05–661, Millipore, Temecula, CA), PAR (#ALX-804-220, Enzo, Farmingdale, NY), PARG (# 66564, Cell Signaling, Danvers, MA), γ-H2A.X (# 2577, Cell Signaling, Danvers, MA), and PARP (#9542, Cell Signaling, Danvers, MA). Membranes were then incubated with the secondary α-mouse (#W402B, Promega, Madison, WI) or α-rabbit (#W401B, Promega, Madison, WI) HRP- conjugated antibodies. Membranes were developed using SuperSignal West Pico ECL (Pierce, IL) and images captured using a Kodak Gel Logic 2000 Imaging System (Molecular Imaging Systems, Rochester, NY). Densitometry profile was analyzed by ImageQuant TL software (GE Healthcare Chicago, IL).

### Immunoprecipitation

LNCaP cells were plated in 10% FBS medium and allowed to adhere overnight. The following day medium was replaced with one supplemented with 10% CSS and either DMSO vehicle or 1 μM PDDX. Cells were harvested and cellular extracts supplemented with 1 μM PDDX and 3 μM ABT-888 to inhibit residual PARG and PARP activity, diluted four-fold with TBS, and immunoprecipitated with 0.4 μg of PARP antibody overnight at 4 °C on a rolling incubator. Immune complexes were adsorbed on Protein A coated magnetic beads (#88845, Thermo Scientific, Rockford IL), washed 3 times with TBS, and eluted with Laemmli buffer at 37 °C. For the western blots, 6% of eluate were used for PARP1 and 50% eluate for PAR detection. Images were captured using a GE ImageQuant LAS 500.

### Cellular proliferation assay

Proliferation was assessed using a Roche DP Real Time Cell Analyzer (RTCA). Background impedance was established after incubating E-plates (Acea Biosciences, San Diego, CA) with 50 µl of medium at room temperature for 30 minutes. LNCaP cells were seeded in 100 µl per well at a density of 1.5 × 10^4^ cells per well in FBS media or 2 × 10^4^ cells in CSS media. LAPC4 cells were seeded in 100 µl per well at a density of 5 × 10^4^ cells per well. The cells attached overnight and then were treated with DMSO vehicle, 1 µM PDDX, 100 µM TMZ, or a combination of 1 µM PDDX and 100 µM TMZ. During the attachment and after treatment with compounds, impedance was measured every 30 minutes. Impedance is represented by cell index (CI) and is calculated as follows: CI = (*Z*_*i*_* − Z*_*o*_)/15 Ω where *Z*_*i*_ is impedance at an individual time point, and *Z*_*o*_ is the background impedance. Average CI was calculated from four wells per treatment at each time point and normalized to the impedance immediately after compound addition, which was assigned a value of 1.

### BER capacity assay

The LNCaP and LAPC4 cells were treated as indicated, scraped in ice-cold PBS, sedimented by centrifugation at 3000 rpm for 10 min, and washed twice with PBS. Cell extracts were made as described previously^65^ and dialyzed into BER reaction buffer containing 50 mM Tris-HCl, pH 7.5, 50 mM KCl, 0.1 mM EDTA, 0.1 mg/ml bovine serum albumin, and 0.01% Nonidet P-40. Radiolabeled substrates (25 nM) that contained a tetrahydrofuran residue (THF), an analog for the abasic site, were pre-incubated with 5 nM APE1 purified as previously described^66^ at 37 °C for 15 min, allowing the substrate to be completely converted SSB intermediates for subsequent BER reactions. BER capacity of LNCaP and LAPC4 cell extracts was measured by incubating the THF containing substrate with 50 μg cell extracts at 37 °C for 30 min in a 25 μl reaction volume in the BER reaction buffer [5 mM Mg^2+^, 50 μM dNTPs, and 2 mM ATP]. The reactions were terminated by adding 25 μl of stopping buffer [95% formamide and 2 mM EDTA] and incubating at 95 °C for 5 min. Repair products were then separated on 15% urea-denaturing PAGE and detected by a Pharos FX Plus PhosphorImager (Bio-Rad Laboratories, Hercules, CA). The percentage of the repaired product was quantified as previously described^38^.

### MTT assay

LNCaP cells were seeded in a 96-well plate at 2 × 10^3^ cells per well in medium supplemented with either FBS or CSS and allowed to adhere overnight. The following day treatment was added to the wells, and the plates were incubated for 48 hours. After incubation, 50 µl of culture media was removed from each well, and 50 µl of MTT (2 mg/ml) (Acros Organics, Atlanta, GA) was added. The plate was then incubated in the dark at 37 °C for 4 hours, 150 µl of the media/MTT solution was replaced with 100 µl of DMSO. Plates were incubated for 15 min and absorbance at 570 nm was measured using a ClarioStar Plate Reader (BMG LabTech, Ortenburg, Germany).

### Statistical analysis

Student t-tests were used to test for inequality of means from two independent samples. P-values less than 0.05 were considered statistically significant. Values are presented as mean ± SEM. Biological triplicates were used for every point in individual experiments for evaluating changes in gene expression.

## Supplementary information

Supplementary Information.

## Data Availability

All datasets analyzed in the current study are publicly available and can be accessed at https://www.ncbi.nlm.nih.gov/geo/. The following gene expression datasets were used in this manuscript: GSE62474, GSE33316, GSE74367, GSE70770, and GSE120716.
