# Supplementary material for: Androgen Receptor and Poly(ADP-ribose) Glycohydrolase Inhibition Increases Efficiency of Androgen Ablation in Prostate Cancer Cells
Source: Sci Rep. 2020 Mar 2;10:3836. doi: 10.1038/s41598-020-60849-y (PMC7052214; doi:10.1038/s41598-020-60849-y)
Supplement: Supplementary file 1 — Supplementary Information. [file 41598_2020_60849_MOESM1_ESM.pdf]

Androgen Receptor and Poly(ADP-ribose) Glycohydrolase Inhibition Increases Efficiency of Androgen Ablation in Prostate Cancer Cells

Manqi Zhang[1], Yanhao Lai[2,3], Judy L Vasquez[4], Dominic I James[5], Kate M Smith[5], Ian D Waddell[5,6], Donald J Ogilvie[5], Yuan Liu[2, 3] and Irina U Agoulnik[3, 4, 7,*]

[1]Biochemistry Ph.D. Program, Florida International University, Miami, FL, USA
[2]Department of Chemistry and Biochemistry, College of Arts, Sciences and Education, Florida International University, Miami, FL, USA.
[3]Biomolecular Sciences Institute, Florida International University, Miami, FL, USA
[4]Department of Human and Molecular Genetics, Herbert Wertheim College of Medicine, Florida International University, Miami, FL, USA.
[5]Cancer Research UK Manchester Institute, The University of Manchester, Alderley Park, SK10 4TG, U.K.
[6]Current address: CRL, Chesterford Research Park, CB10 1XL, UK
[7]Department of Cellular and Molecular Biology, Baylor College of Medicine, Houston, TX.

***Corresponding Author:** Irina U. Agoulnik, PhD, Department of Human and Molecular Genetics, Herbert Wertheim College of Medicine, Florida International University, 11200 SW 8th Street, Miami, FL 33199, USA. Irina.Agoulnik@fiu.edu. Phone: 305-348-1475, Fax: 305-348-0688

Gene	Forward	Reverse	Probe
18S	gcaattattccccatgaacg	gggacttaatcaacgcaagc	48
AR	gccttgctctctagcctcaa	gtcgtccacgtgtaagttgc	14
INPP4B	tgtctgatgctgacgctaaga	ccacaaaccaatccagcaa	41
PARG	tctgccaatcacacagtaactattc	taatgtgttggaaaaggtttagga	2
TMPRSS2	ctgatcacaccagccatgat	tatcccctatcagccaccag	4
UGT2B17	tgtcagaagaaagtgccaaca	gccatcaaatctccatagaacc	61
PARG	aaagcgcattacttcaaaaactg	gcaagtcaggatccaagagaa	16
***PSA* promoter**	cccctgtttctgtttcatcc	gccctgtagctcatggagac	82

Supplemental Table 1. qPCR primer sequences and corresponding probes used to analyze gene expression.

Supplementary Figure S1

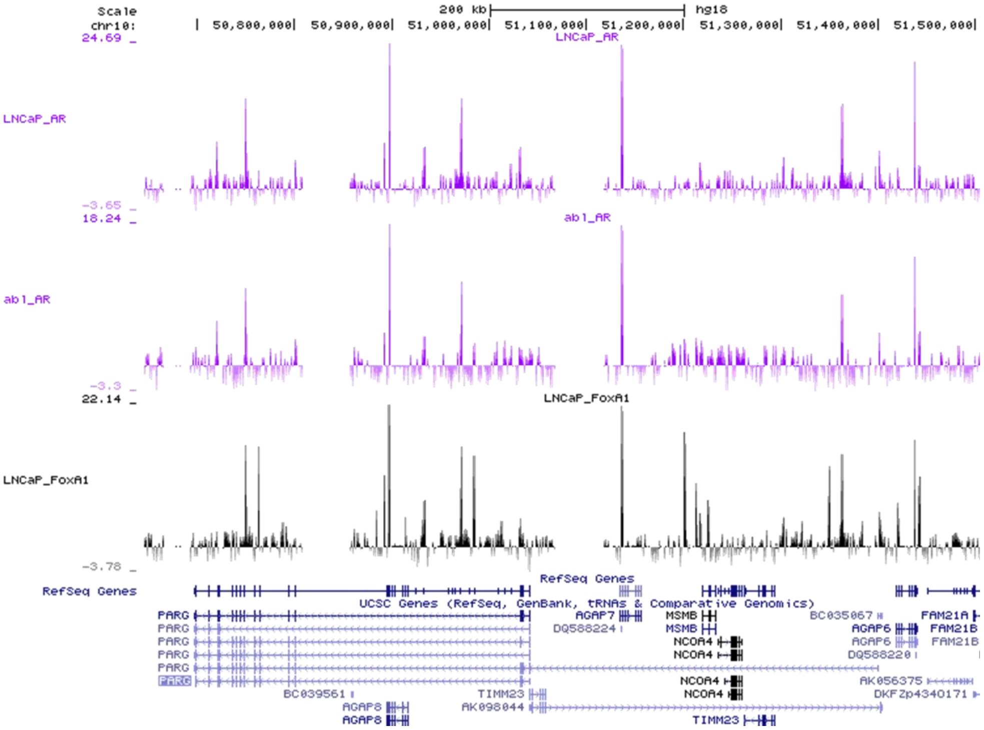

Supplementary Figure S1. Recruitment of androgen receptor (AR) and pioneer factor FOXA1 to the *PARG* locus in androgen-dependent prostate cancer cell line LNCaP and its castration-resistant derivative cell line, LNCaP-abl. Below are the various splice variant transcripts of *PARG* and transcripts of adjacent genes.

Supplementary Figure S2

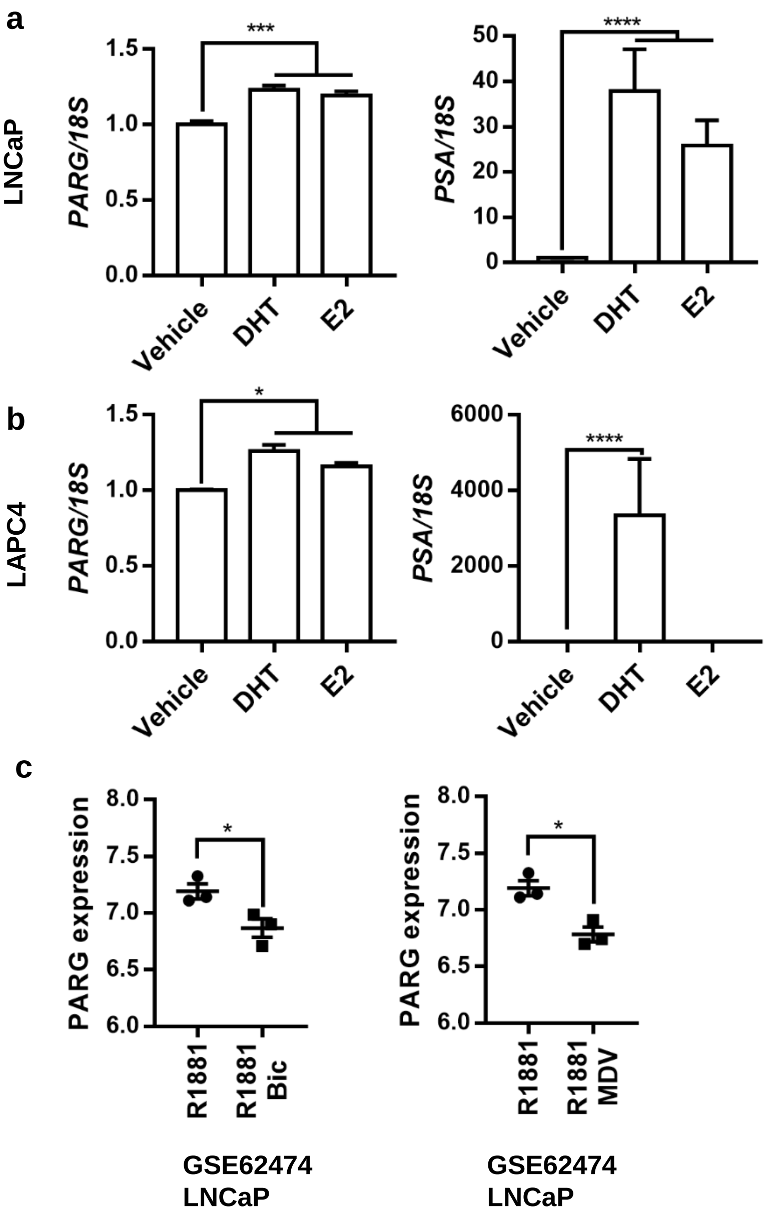

Supplementary Figure S2. LNCaP (**a**) or LAPC4 (**b**) cells were placed in medium supplemented with 10% CSS. Next day cells were treated with 100 nM dihydrotestosterone (DHT) or 100 nM estradiol (E2) as indicated for additional 72 hours. RNA was analyzed for expression of *PARG* and *PSA*. Expression was normalized to *18S*. Each bar is an average of 3 biological replicates. The experiments were performed 3 times. (**c**) *PARG* expression in LNCaP cells treated 24 hours with R1881 or R1881 plus bicalutamide (Bic, left) or enzalutamide (MDV, right) for 24 hours. Data were acquired from GSE62474.

Supplementary Figure S3

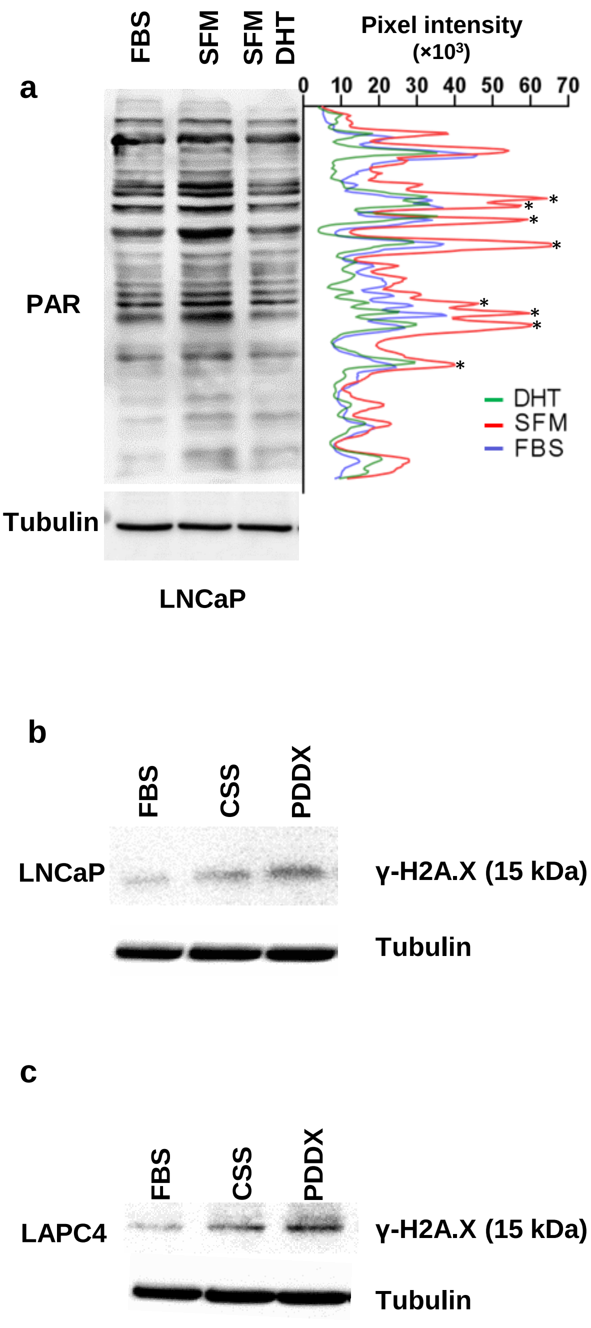

Supplementary Figure S3. A. LNCaP cells were placed in RPMI medium with 10% FBS or serum-free medium (SFM). Cells were treated with vehicle or 10 nM DHT as indicated for 5 days. Protein levels of PAR and tubulin were assessed by Western blotting and the densitometry profile of PAR is shown. Three measurements of signal intensities were performed along each line and averaged and normalized to signal intensity of the tubulin band. PAR intensities that were significantly changed from FBS control ($p<0.05$) were marked with an asterisk *. The experiment was performed 3 times.

Supplementary Figure S4

PDD00017272 Properties	
**Structure**	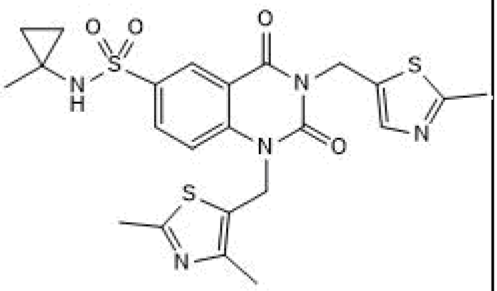
**Solubility in PBS + 1% DMSO (μM)**	**5.2**
**Solubility in DMSO (mM)**	**20**
**PARG Biochemical EC50 (μM)**	**0.0048**
**PARG Cell EC50 (μM)**	**0.0092**
**Cytotoxicity (μM) (HeLa)**	**>30**
**Lipophilicity (XLogP)**	**4.7**
**Intrinsic Clearance CL_{int} (μL/min/mg) (human hepatocytes)**	**135**
**Half Life (human hepatocytes)**	**10 min**

Supplementary Figure S4. Biochemical properties of PDD00017272

Supplementary Figure S5

Figure 1

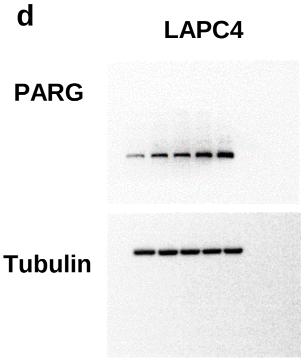

Figure 1

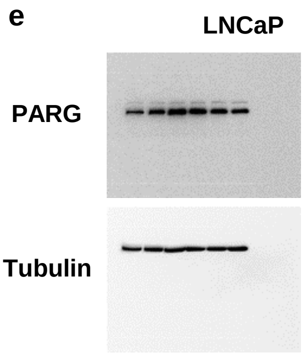

Figure 2

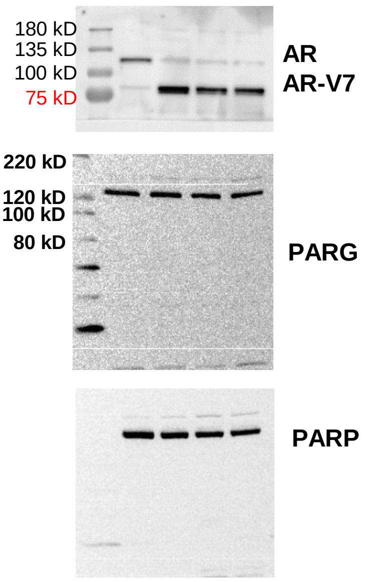

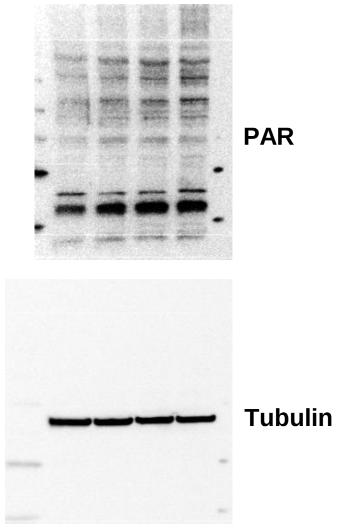

Figure 3

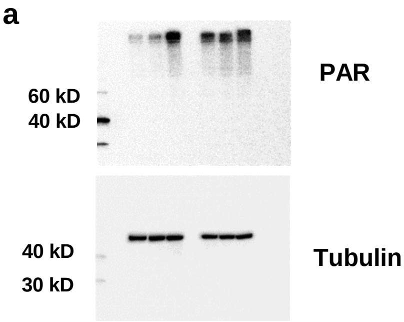

Figure 3

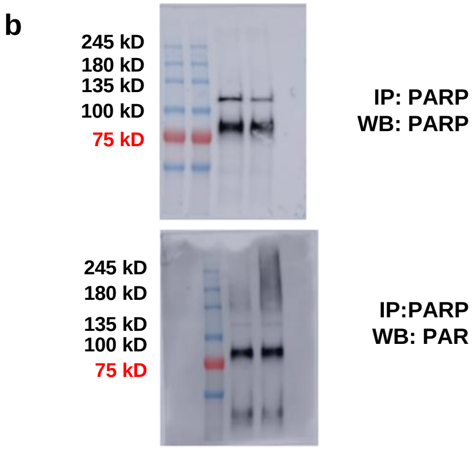

Supplementary Figure S5 continue

Figure 5

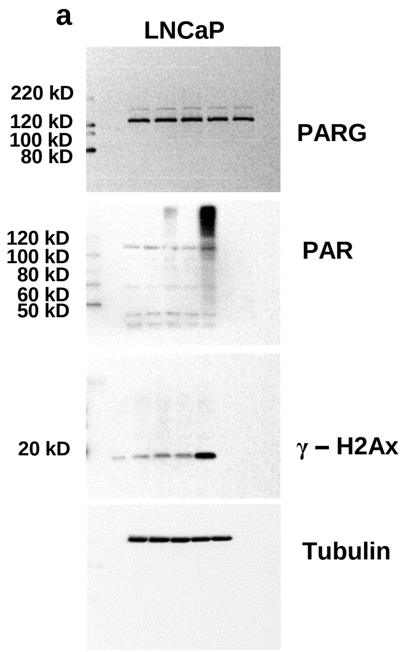

Figure 5

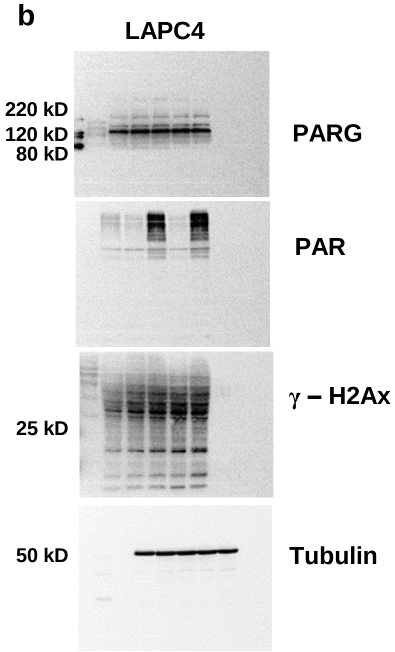

Supplementary Figure S5. Full length images of cropped blots presented in Figures 1, 2, 3 and 5.

Supplementary Figure S6

Abbreviation	Definition
APE1	Apurinic/pyrimidinic endonuclease 1
AR	Androgen receptor
ARH1	ADP-Ribosylarginine Hydrolase 1
ARH3	ADP-Ribosylarginine Hydrolase 3
BER	Base excision repair
ChIP Assay	Chromatin ImmunoPrecipitation Assay
CRPC	castration-resistant prostate cancer
CSS	Charcoal Stripped Serum
DSB	double stranded breaks
ERG	ETS Transcription Factor
FBS	Fetal Bovine Serum
FoxA1	Forkhead Box A1
KDM4D	Lysine Demethylase 4D
LIG III	DNA Ligase 3
PAR	poly ADP-Ribose
PARG	Poly(ADP-ribose) glycohydrolase
PARP	poly(ADP-ribose) polymerase
Pol β	polimerase β
RAR	Retinoic Acid Receptor
SSB	Single Strand Break
TMPRSS2	Transmembrane Serine Protease 2
TMZ	temozolomide
UGT2B17	UDP Glucuronosyltransferase Family 2 Member B17
XRCC1	X-Ray Repair Cross Complementing 1
γH2A.X	Phosphorylated form of H2A histone family member X

Supplementary Figure S6. Abbreviations.
